# Temporally Variable Geographical Distance Effects Contribute to the Assembly of Root-Associated Fungal Communities

**DOI:** 10.3389/fmicb.2016.00195

**Published:** 2016-02-25

**Authors:** Christopher J. Barnes, Christopher J. van der Gast, Caitlin A. Burns, Niall P. McNamara, Gary D. Bending

**Affiliations:** ^1^School of Life Sciences, Gibbet Hill Campus, University of WarwickCoventry, UK; ^2^Section of Evolutionary Genomics, National History Museum of Denmark, University of CopenhagenCopenhagen, Denmark; ^3^Natural Environment Research Council Centre for Ecology and HydrologyWallingford, UK; ^4^Natural Environment Research Council Centre for Ecology and Hydrology – Lancaster Environment CentreLancaster, UK

**Keywords:** fungal ecology, mycorrhizal fungi, root-associated fungi, soil fungi, temporal variation in microbial communities

## Abstract

Root-associated fungi are key contributors to ecosystem functioning, however, the factors which determine community assembly are still relatively poorly understood. This study simultaneously quantified the roles of geographical distance, environmental heterogeneity and time in determining root-associated fungal community composition at the local scale within a short rotation coppice (SRC) willow plantation. Culture independent molecular analyses of the root-associated fungal community suggested a strong but temporally variable effect of geographical distance among fungal communities in terms of composition at the local geographical level. Whilst these distance effects were most prevalent on October communities, soil pH had an effect on structuring of the communities throughout the sampling period. Given the temporal variation in the effects of geographical distance and the environment for shaping root-associated fungal communities, there is clearly need for a temporal component to sampling strategies in future investigations of fungal ecology.

## Introduction

Root-associated fungi are functionally and genetically diverse, and can play an integral role in connecting the aboveground biomass to the belowground ecosystem (Berendsen et al., 2012). Root-associated fungi can be both beneficial and detrimental for plant growth. Mycorrhizal fungi are obligate root symbionts, which can dominate the root-associated microbial biomass (Högberg and Högberg, 2002) and are associated with enhanced plant nutrient uptake (Garcia et al., 2014) and disease resistance (Chakravarty and Unestam, 1987; Liu et al., 2007). Similarly, saprophytic fungi decompose organic matter, thereby promoting nutrient availability to plants (Niklaus et al., 2001). Conversely, phytopathogenic fungal species also reside within the rhizosphere, causing disease and reducing plant growth (Espinoza et al., 2008). Given the strong and variable effect the root-associated fungal community can have on aboveground biomass, and the potential for shifts in root-associated fungal composition to influence ecosystem function (Berg and Smalla, 2009; Smith and Read, 2010), the understanding of how these communities assemble is of significant ecological importance yet remains relatively poorly understood.

Plant hosts have been shown to play a major role in determining rhizosphere fungal communities. The main types of mycorrhizal fungal associations are generally confined to specific groups of plant (Smith and Read, 2010), and individual mycorrhizal fungal species can associate with broad or narrow ranges of plant species (Allen, 1992; Lang et al., 2010), and even genotypes within a species (Leski et al., 2010). There is also a large body of literature linking root-associated fungal community assembly with edaphic properties, with the composition of ectomycorrhizal (ECM), arbuscular mycorrhizal (AM) and non-mycorrhizal fungi all shown to be associated with specific soil parameters (Berg and Smalla, 2009). Whilst soil pH in particular is almost ubiquitously important for determining soil microbial community composition and function (Högberg et al., 2001; Toljander et al., 2006; Griffiths et al., 2011), soil nutrients such as P, K, N, and Ca have also been shown to influence root-associated fungal community structure (Jones et al., 1990; Lilleskov et al., 2002; Högberg et al., 2010; Põlme et al., 2013). The high nutrient concentrations and reduced pH that often occur following regular fertilization within agricultural soils can have particularly profound effects on root-associated fungi, leading to reduced richness and biomass (Phillips and Fahey, 2007; Verbruggen et al., 2012; Gosling et al., 2013).

The composition of root-associated fungal communities has also been shown to vary temporally, with seasonal changes in the composition of ECM (Jumpponen et al., 2010), AM fungi (Dumbrell et al., 2011), saprophytes and pathogens (Hilton et al., 2013). However, there is much uncertainty about the factors which drive temporal changes in root-associated fungal composition, and furthermore the extent to which environment and time interact to affect community composition remains to be elucidated (Last et al., 1984; Pickles et al., 2010). Abiotic factors such as climatic variables (Swaty et al., 1998; Bahram et al., 2012), and biotic factors such as plant growth and vegetation composition vary throughout the year, and these have been hypothesized to drive changes in root-associated fungal composition over time (Dumbrell et al., 2011). However, ecological drift, in which stochastic processes contribute to heterogeneous distributions of taxa in time, could also play a role in shaping changes in root-associated communities over time (Martiny et al., 2011). A major area of uncertainty is the extent to which root-associated fungi, and microbial communities in general, show true seasonality through the formation of distinct and predictable seasonal assemblages, or simply undergo ecological drift, since comprehensive studies over long-time periods have scarcely been performed.

The way in which geographical distances between sampling locations affects root-associated community assembly has become an area of increasing interest in recent years, with the magnitude of effects varying greatly between geographical scales and systems that have been sampled. For AM fungi, strong evidence of dispersal limitation was found at the continent scale, however, this disappeared at environmental extremes (Green et al., 2004; Kivlin et al., 2011). Other studies have found limited effects of geographical distance on AM community composition at the regional level (An et al., 2008; Peay et al., 2010; van der Gast et al., 2011; Hazard et al., 2013), although spatial scaling effects at the local level may have been overlooked through ‘pooling’ of samples from the same sampling site. Whilst the role of distance in determining ECM communities has been studied less than AM communities, previous investigations found have found evidence of spatial scaling effects within ECM communities at the local level (Lilleskov et al., 2004), with effects also shown to be much greater at larger spatial scales, with distributions of ECM fungi reflecting barriers to dispersal at the regional and global levels (Peay et al., 2010; Bahram et al., 2012; Põlme et al., 2013). Given the repeated findings of a breakdown in community similarity within fungal communities at the regional and global spatial scales (Kivlin et al., 2011), and less frequently at the local level, the ubiquitous dispersal hypothesis (Finlay and Clarke, 1999) which proposes that microbes have a cosmopolitan distribution seem unlikely to hold true. More recently Foissner described a model of ‘moderate endemicity’ for protists, where some species have cosmopolitan distributions, and others are dispersal limited (Foissner, 2006, 1999), a model which could prove useful in describing the spatial distribution of other microbial communities, including root-associated fungi.

In the current study we quantified the influence of geographical distance between root-associated fungal communities and soil characteristics on structure of root-associated fungal communities over a 13-month period. Using a commercial short rotation coppice (SRC) willow plantation in the UK, roots were collected over line transects within the site, at four time points between October 2010 and October 2011. Terminal restriction length polymorphism (TRFLP) was supplemented with 454-pyrosequencing to assess variation of fungal community composition in space and time.

## Materials and Methods

### Study Site and Experimental Design

Biomass energy crops are monodominant-cropping systems that remain untilled throughout their lifetime, thus allowing microbial communities to develop over time in a relatively undisturbed soil matrix in a low-diversity aboveground system. This study used a SRC willow plantation at a field site near Lincoln, Lincolnshire, UK. The soil was a fine loam over clay, with approximately 15% clay, 49% sand, and 36% silt. The 30-year mean air temperature was 9.9°C. Soil at the site has a pH gradient that ranged from 5.2 in the southern edge of the site to 6.8 in the northernmost, and has a mean total C and N content of 1.81 and 0.28% respectively. The willow was planted in 2000 at a density of 15,000 stools ha^-1^ and covered approximately 9.44 ha. Previously the land was rotated between wheat and oilseed rape for at least 20 years before conversion to willow. Trees were planted in paired rows, with trees within rows planted roughly 0.75 m apart, whilst neighboring trees between paired rows were 1.5 m apart. The willow consisted of 6 different varieties of *Salix viminalis* that were planted according to commercial practice to prevent disease spread, with Tora (60%) being the most abundant (the others being Bjorn (10%), Bowles Hybrid (10%), Jorr (10%) and Jorunn (10%)). The crop was coppiced first in 2001, then in 2004, 2007, and 2010 with an average annual yield of 6.72 t ha^-1^. The growing season is between March and September for SRC willow in the UK. The site received concentrated PK fertilizer (Fibrophos, UK) at 660 kg ha^-1^ at establishment, with 20 tones of compost and lime applied to the field site in February 2010. No further exogenous nutrient input was applied during the course of the experiment.

**FIGURE 1:**
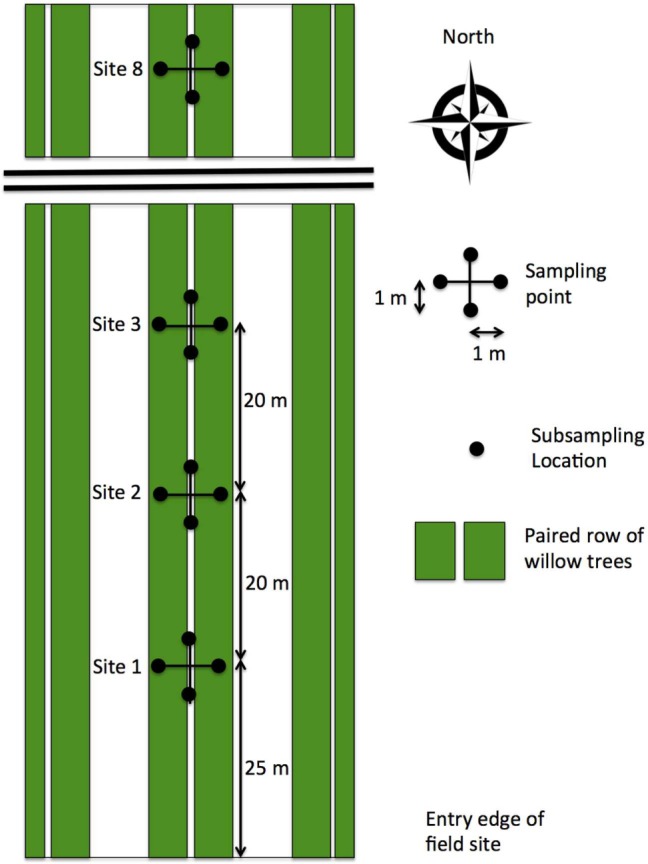
**Schematic of the line transects.** Transects were sampled due north along a single paired row of the willow trees, starting 25 m into the site to avoid edge effects. Sampling locations were 20 m apart. Sampling locations consisted of 4 cm × 15 cm deep soil cores taken at 1 m distances around the central point.

A single row of the willow was used for line transects, which started from the southern edge of the field heading north. In order to avoid edge effects, transects started 25 m along the row, and eight locations were sampled every 20 m across the row, spanning 160 m (**Figure 1**). Subsequent transects shifted sequentially 3 m north of the previous sampling in order to avoid repeat sampling of disturbed soil. At each location, four subsamples were taken in a cross shape 1 m around a central point. These consisted of 4.5 cm diameter soil cores that were 15 cm deep (van Walt, Netherlands). Transects were taken in October 2010, and July, August, and October 2011.

### Soil Nutrient Analysis

For mineral analysis, 100 g of soil was air dried for 1 week before being ground and sieved to <2 mm particle size. To measure pH, 10 ml of soil was added to 25 ml deionized water and the mixture shaken for 15 min before measurement using a pH meter (Accumet AB15, Fischer Scientific, UK). In order to measure available P, 5 g of finely ground soil was shaken for 30 min in 0.5 M NaHCO_3_, before analysis on an inductively coupled plasma optical emission spectrometer (ICP) (Jobin-Yvon ICP-OES, HORIBA, UK; Olsen, 1954). Available K and Mg were extracted by shaking 10 g of soil with 1 M NH_4_NO_3_ for 30 min, followed by centrifugation. Concentrations of K and Mg in the supernatant were measured using ICP (Bremner and Keeney, 1965). NH_4_, and NO_3_ were extracted by shaking 20 g of soil with 10.5% K_2_SO_4_ for 2 h, and following centrifugation, measured in the supernatant using a FIAstar 500 flow injection analyser system (FOSS, Denmark) (Bremner and Keeney, 1965; Henriksen and Selmer-Olsen, 1970).

### Sample Preparation

Soil was softened by soaking at 19°C for 1 h in deionized water, before roots were hand extracted using forceps. Non-senescent fine roots were selected by morphology (lighter color and branched structure with the presence of fine root tips). Roots less than 2 mm diameter were washed on a 6 mm sieve in order to remove adhering soil. Roots were then cut into 1 cm lengths and mixed thoroughly, before 0.5 g was taken for DNA extraction. Using the lysis tubes provided with the PowerSoil DNA isolation kit (MP Biomedicals, Cambridge, UK), roots were mechanically lysed in a TissueLyser (QAIGEN, UK) using three separate 30 s pulses at 30 Hz, before undergoing the remainder of the extraction process exactly as per manufacturers instructions.

### TRFLP Protocol

Labeled ITS1F (5-CTTGGTCATTTAGAGGAAGTAA-3)-6FAM and ITS4 (5-TCCTCCGCTTATTGATATGC-3′)-TET primers were used to investigate root-associated fungal communities (Gardes and Bruns, 1993). PCR was performed using 25 ng of DNA from each individual subsample in a total volume of 50 μl, which included 47 μl of Megamix (Microzone, Haywards Heath, UK), 1 μl of forward and reverse primers, and 1 μl of 25 ng μl^-1^ template DNA from samples. The program for PCR consisted of: 5 min at 92°C; followed by 25 cycles of 30 s at 92°C, 90 s at 56°C, followed by 30 s at 72°C; a final extension of 5 min at 72°C.

ITS amplicons from each subsample underwent digestion with *Hpy8I* (Fermentas, UK). 20 μl reactions contained 200 ng of DNA, 2 units of enzyme and 2 μl of x10 manufacturer’s buffer before reactions were equilibrated to 20 μl using molecular grade water (MO BIO, Carlsbad, CA, USA) and incubated at 37°C overnight. Following digestion, samples were run through sephadex columns for further purification (Sigma–Aldrich, Germany). Four micro liter of digested and purified samples were then loaded on a capillary sequencer (ABI 3010, Applied Biosystems, UK). Samples were run with GeneScan 1200 LIZ ladder (Applied Biosystems, UK).

Genemarker v1.50 (Softgenetics, State College, PA, USA) was used to quantify the number and area of the resulting TRFLP peaks, which were exported to MS Excel (Microsoft, USA) for further analysis. Baseline noise was considered to be 50 fluorescence units, with peaks lower than this removed from the analysis. Samples were normalized by the conversion of peak areas (independently for the 5′ and 3′ ends) to percentage relative abundance of the total fluorescence area (Hilton et al., 2013).

### Barcoded Pyrosequencing

The 4 subsamples collected in October 2010 at each sampling location were equilibrated to 25 ng μl^-1^ and 10 μl of each was pooled to make a DNA template from each of the eight sampling locations for pyrosequencing. In order to improve comparability with the data produced via TRFLP, unlabeled ITS1F and ITS4 primers were used to generate pyrosequencing data. However, it should be noted that ITS1F and ITS4 primers show some selectivity (De Beeck et al., 2014) and produce relatively long fragments that can increase bias in community analyses (Ihrmark et al., 2012) when used in high-throughput sequencing. The initial PCR with the ITS primers was performed with MyTaq HS DNA polymerase (Bioline, USA) and consisted of: 1 μl of DNA template, 2 mM dNTPs and 10 pmol of each primer. Thermocycler conditions were 95°C for 5 min; 40 cycles of 95°C for 30 s, 55°C for 30 s, 72°C for 0.5 min; and 72°C for 5 min, using a Biometra TJ3000 thermocycler (Biometra, Germany). A secondary semi-nested PCR reaction was performed to add the fusion primer necessary for pyrosequencing. Fusion primers consisted of: GS FLX LR70-specific adapter A, a multiplex identifier (MID), and a new forward primer, a modified version of the universal M13 primer. The fusion primers used in the secondary reaction were: forward 5′- *GTGTGAAATTGTTACGCT* (10-bp MID) CTTGGTCATTTAGAGGAAGTAA-3 and reverse 5′ TCCTCCGCTTATTGATATGC-3′. The forward primer comprised of the A adapter (in italic type) for the pyrosequencing reaction, the 10-bp MID is part of Roche’s extended MID set (www.454.com) and the final part is the modified M13 primer. The reverse primer consists of the fusion adapter B only. The secondary PCR was performed in a volume of 25 μl, and consisted of: 1 μl of DNA template, 2 mM dNTPs and 10 pmol of each primer. Thermocycler conditions were: 94°C for 1 min 40 s; 40 cycles of 95°C for 20 s, 55°C for 20 s and 72°C for 20 s; and 95°C for 10 min. Sample concentrations were calculated by SYBR gold based quantitation (Shmidazu, Japan), before two plates of 20 equimolar concentrations of MID tagged samples were loaded onto a Roche 454 GS Junior pyrosequencer (454 Life Sciences/Roche Applied Biosystems, Nurley, NJ, USA) and sequenced at Micropathology Ltd (Coventry, UK). Raw sequence reads were deposited at the NCBI Sequence Read Archive under the accession number **SRR1951015**.

### Processing of Pyrosequencing Data

Sequencing data underwent denoising with Acacia-1.52 software (Bragg et al., 2012). The software package ‘Quantitative insights into microbial ecology’ (QIIME v1.7.0, USA) was used to perform the majority of the remaining sample processing (Caporaso et al., 2010). OTUs were picked *de novo* using the UCLUST algorithm at 97% similarity and from these reads a representative sequence set was created from across all samples (Edgar, 2010). Chimeric sequences were removed using the UCHIME algorithm in *de novo* mode (as part of USEARCH8.0; Edgar et al., 2011). Taxonomy was assigned at the 97% level using the 27.08.2013 release of the ITS fungal database for QIIME, from the UNITE project, before an OTU table was created with the taxonomic assignment and relative amplicon abundance for each sample (Wang et al., 2007). An initial 28,125 sequences formed 2,138 OTUs. Samples were subsampled to the lowest sample number, 890, before the removal of singletons, leaving approximately 592 reads per sample. In total, there were 4,738 reads spanning 320 OTUs. A second round of taxonomic assignments were performed on OTUs of greater than 1% relative abundance in which sequences underwent manual BLAST searches against the constantly updated online version of the UNITE database to increase taxonomic resolution (**Table 3**). Taxonomy was reassigned when sequence identity was 99% or greater and a DOI was added to improve data reproducibility.

### Statistical Analyses

Relative abundance data produced via TRFLP underwent arcsine transformation in order to homogenize variation before statistical analyses were performed. To compare differences in alpha-diversity, the number of TRFs [terminal restriction fragment(s)] between sampling locations and sampling time points were analyzed using repeated measures analysis of variance (ANOVA). Beta diversity was assessed by calculating distance decay-rates (DDR) across each transect as previously described (Green et al., 2004). Briefly, Bray–Curtis similarity matrices were created from the community data and Euclidean distance matrices were created from the distance between individual subsamples, using the vegdist package within R (Bray and Curtis, 1957). An exponential gradient was calculated by plotting the similarity values of the community against geographical distance, giving the distance-decay rate (using the formula *S* = *cD*^ddr^). Differences between the rates of decay between sampling points were assessed using the *t*-distribution method (Fowler et al., 1998).

For direct ordination, community data underwent arcsine transformations before undergoing canonical correspondence analysis (CCA) with integrated forward selection (Canoco v5.0, Wageningen, Netherlands; Braak and ter Šmilauer, 2002). In order to incorporate distance between sampling locations within the CCA, principle coordinates of neighbor matrices (PCNM) were calculated from grid coordinates for each subsample and the initial first PCNM was used as an explanatory variable (using the Vegan package of R). Forward interactive selection was performed to obtain significantly correlating explanatory variables, including soil properties (pH, C, N, NO_3_, NH_4_, Mg, P, and K) and geographical separation (as PCNM1) against the ordination analysis of the community, whilst limiting the effects of multicolinearity (van den Wollenberg, 1977). The total variation explained in the ordination analysis as well as the variation explained by each explanatory variable was calculated during the analysis.

To investigate temporal variation in community composition, the TRFLP datasets were used to generate a single Bray–Curtis matrix. A non-metric multidimensional scaling (nMDS) analysis was performed and used to visualize community similarity between the different seasons. In order to partition and quantify the temporal variance explained by and time (in weeks after sampling started), the ADONIS function in the Vegan package of R, using the combined community Bray–Curtis matrix and sampling time points as the main factor (either October 2010, July 2011, August 2011, or October 2011) was used. As this analysis is sensitive to the order explanatory variables are analyzed, individual ADONIS analyses were performed for each month (either October, July, August) and time (in weeks after sampling started) and placed in order of largest proportion of variation explained before a combined analysis was performed.

To compare community profiles produced using TRFLP and 454 pyrosequencing approaches, using the October 2010 dataset a paired *t*-test was performed between the average number of TRF at each sampling location and the number of OTU determined using pyrosequencing. In addition a DDR was calculated for the pyrosequencing dataset as described above, and this was compared to the DDR curve produced using TRFLP data, using the *t*-distribution method, as used previously.

## Results

### Edaphic Properties

**FIGURE 2:**
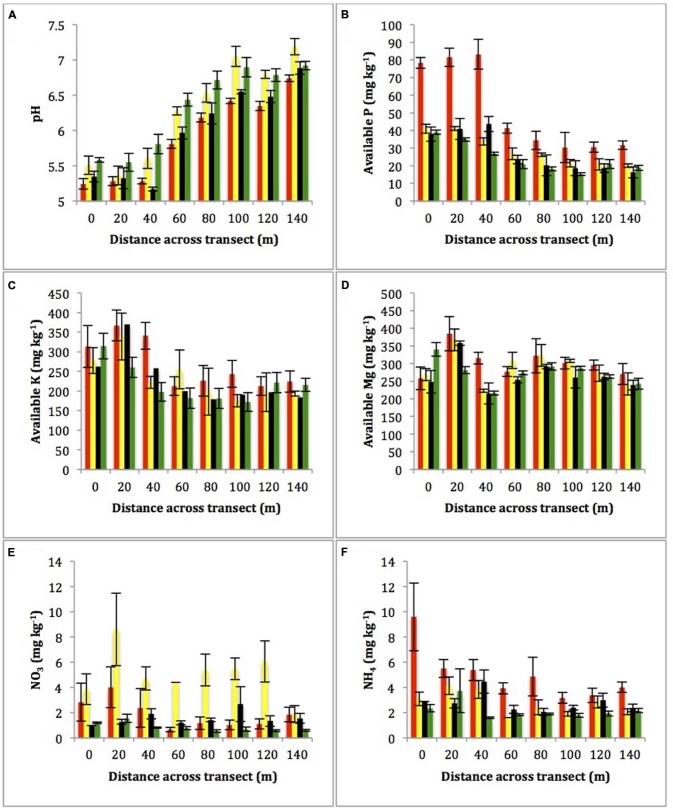
**Average of the four subsamples per sampling location of (A) pH, (B) available P (mg kg^-1^), (C) available K (mg kg^-1^), (D) available Mg (mg kg^-1^), (E) NO_3_ (mg kg^-1^), and (F) NH_4_(mg kg^-1^) across the transects**. Error bars are ± 1 SEM.

There were strong gradients in pH, available P and available K across transects, which were conserved across all seasons (**Figures 2A–C**), with P (*r* = 0.687, *P* < 0.001) and K (*r* = 0.573, *P* < 0.001) inversely correlating with pH. pH ranged from 5.2 to 6.8 across the 160 m transect in all seasons, while available P varied from 80 mg kg^-1^ to 35 mg kg^-1^ across the transect in October 2010, and from 40 to 20 mg kg^-1^ in the following seasons. Similarly available K was present at over 300 mg kg^-1^ in transect sites 1–3 and at approximately 200 mg kg^-1^ in sites 5–8 across all time points. The available Mg concentration did not significantly change over the length of transects or over time (**Figure 2D**). Both NO_3_ (*P* < 0.001) and NH_4_ demonstrated significant (*P* < 0.001) variation over time, with highest concentrations in the October 2010 and July 2011 transects respectively (**Figures 2E,F** respectively). Whilst NH_4_ ranged from 1.59 to 9.59 mg kg^-1^, no significant differences were found between sampling locations, NO_3_ ranged from 0.54 to 8.60 and significantly varied between sampling locations (*P* < 0.001).

### Fungal Community Analysis

From the root-associated fungal TRFLP analysis, 273 different TRFs were identified over the four seasons, with TRF richness ranging between 22.0 and 83.8 across sampling locations. TRF richness significantly varied between sampling locations (*P* = 0.023), with site 4 (60 m into the transect) consistently having a lower TRF richness than the other sites. The number of TRFs did not differ between sampling times (*P* = 0.058).

**FIGURE 3:**
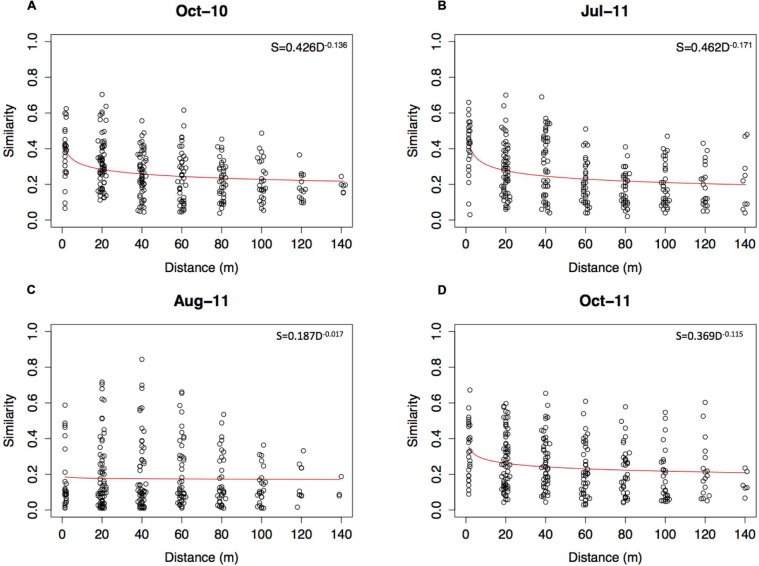
**Distance-decay of fungal community similarity over the (A) October 2010 (B) July 2011, (C) August 2011 and (D) October 2011 transects.** Plotted are the Bray–Curtis similarity values against geographical distance for all paired sampling combinations, with the DDR calculated for each transect using the formula *S* = *cD^ddr^*.

Distance decay-rates were calculated to quantify the breakdown in community similarity of the root-associated fungi across each transect. All seasons showed some decline in similarity over transects, with the DDR varying throughout the sampling period, from -0.136 (*P* = 0.001) in October 2010, to -0.171 (*P* = 0.001), -0.017 (*P* = 0.001) and -0.115 (*P* = 0.001) in July 2011, August 2011 and October 2011 respectively (**Figure 3**). The August 2011 transect had a significantly lower rate of decay across the transect than all other sampling points (*P* = 0.001, *P* = 0.001 and *P* = 0.001 for October 2010, July 2011, and October 2011 respectively), whilst none of these others were significantly different to each other (**Supplementary Table S1**).

### Investigating Distance and Edaphic Effects on the Root-Associated Fungal Community

Canonical correspondence analyses were performed on the TRFLP data with integrated forward selection of explanatory variables (**Table 1**). Soil pH was associated with composition in every season except October 2011 (October 2010 *P* = 0.002, July 2011 and *P* = 0.002, August 2011 *p* = 0.01), accounting for between 7.1 and 9.2% of the variation in the root-associated fungal community. The geographical separation (as PCNM1) across the transect significantly correlated with community variation in both October 2010 (*P* = 0.002) and October 2011 (*P* = 0.002), accounting for 4.8 and 8.4% of community variation.

**Table 1:** Canonical correspondence analysis determining the variation of the rhizosphere fungal communities from TRFLP of each transect explained by metadata parameters.

	Oct-10			Jul-11			Aug-11			Oct-11		
				
Parameter	Var. Exp. (%)	pseudo-*F*	*P*-value	Var. Exp. (%)	pseudo-*F*	*P*-value	Var. Exp. (%)	pseudo-*F*	*P*-value	Var. Exp. (%)	pseudo-*F*	*P*-value
pH	8.5	2.7	0.002	9.2	2.7	0.002	7.1	1.8	0.01	–	–	–
Distance (as PCNM1)	4.8	1.6	0.004	–	–	–	–	–	–	8.4	2.6	0.002
Total	13.3			9.2			7.1			8.4		


*Abbreviations: Var. Exp. (%), the percentage of rhizosphere fungal community variation explained by a parameter given by the canonical correspondence analysis.*

### Investigating Temporal Changes in the Structure of the Root-Associated Fungal Community

**FIGURE 4:**
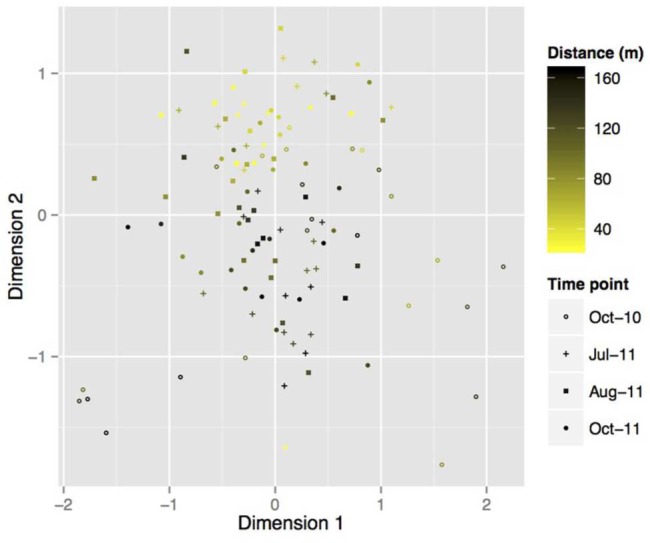
**Two-dimension nMDS plot of the TRFLP community data from all seasons combined, with each point representing a subsample.** Season of origin are differentiated by marker shape whilst color illustrates the distance across the length of the transect sample originated from.

An initial nMDS was performed in order to visualize changes in community composition over time (**Figure 4**). In order to test for significance and quantify temporal changes in community variation, ADONIS analyses were performed to analyse the variance between month of sampling (July, August, and October) and time (in weeks after sampling). Both the month of sampling and time independently accounted for change within the community (**Table 2**), with month of sampling accounting for 7.16% of change with the root-associated fungi (*P* = 0.014) and time 1.53% (*P* = 0.014).

**Table 2:** ADONIS analysis of intra-annual (seasonal) and inter-annual (time in weeks after sampling started) variation within the root-associated fungi of the SRC willow between October 2010 and October 2011.

Parameter	Df	*F*-value	*R*^2^	*P*-value
Season (as either October, July, and August)	2	4.351	0.07158	0.014
Time (in weeks after sampling commenced)	1	1.8652	0.01534	0.014
Residuals	111		0.91308	
Total	114			


### 454-Pyrosequencing Assessment of the October 2010 Community

The pooled DNA stocks for each of the eight sampling locations of the October 2010 transect underwent pyrosequencing to assess the diversity of root-associated fungi. All reads that could be assigned to a phylum were from the Basidiomycota and Ascomycota, with Basidiomycota the dominant fungal phylum, averaging 68.02% of reads and Ascomycota 6.90% of reads from October 2010. Basidiomycota were also more diverse than the Ascomycota, with an average of 41.75 and 9.38 OTUs respectively across the October 2010 community.

OTUs were assigned to the highest taxonomic classification and investigated further. The OTUs consisted of a few dominant and many rare OTUs, with only 15 OTUs having an average abundance greater than 1% (**Table 3**). Reads assigned to a Sebacinales OTU were the most abundant, accounting for 15.00% average abundance, whilst a *Cortinarius diasemospermus* OTU was the second most abundant accounting for a further 10.75% average community abundance. Whilst 4 of the 10 most abundant OTUs were assigned within the *Cortinarius* genus, a further 2 were assigned within the Sebacinales order and another 2 to the Thelephoraceae. Literature suggests that up to 10 of the 15 highly abundant OTUs could be ectomycorrhizal association forming species whilst the Thelephoraceae may also fulfill saprotrophic roles (Matheny et al., 2006; Hibbett, 2007). Reads were also assigned to other potential saprotrophs including *Exophiala equina* and *Didymella exigua*, whilst *D. exigua* may also be pathogenic along with the *Seimatosporium obtusum* OTU (Tanaka et al., 2011; Tedersoo et al., 2014).

**Table 3:** List of the OTUs produced with pyrosequencing of the October 2010 root associated fungal communities.

DOI	Accession number	Taxonomic assignment	Likely habitat	Average abundance
10.15156/BIO/SH180002.07FU	FJ553298	*Sebacinales* sp.1	ECM	15.00
10.15156/BIO/SH188470.07FU	GU817073	*Cortinarius diasemospermus* var. *leptospermus*	ECM	10.75
10.15156/BIO/SH180021.07FU	AJ875375	*Sebacina* sp.1	ECM	6.04
10.15156/BIO/SH186050.07FU	JF519283	Trechisporales sp.	Unknown	5.61
10.15156/BIO/SH218870.07FU	JQ724039	*Hymenogaster griseus*	ECM	5.54
10.15156/BIO/SH188476.07FU	DQ102671	*Cortinarius saniosus 1*	ECM	4.79
10.15156/BIO/SH188572.07FU	AY669621	*Cortinarius saniosus 2*	ECM	4.33
10.15156/BIO/SH188475.07FU	UDB016062	*Cortinarius parvannulatus*	ECM	3.42
10.15156/BIO/SH177909.07FU	UDB008894	Thelephoraceae sp. 1	ECM/Saprotroph	3.14
10.15156/BIO/SH186594.07FU	GU237794	*Didymella exigua*	Saprotrophic/Pathogenic	2.48
10.15156/BIO/SH197643.07FU	JF747094	*Exophiala equina*	Saprotrophic	2.11
10.15156/BIO/SH180002.07FU	HQ211712	Sebacinaceae sp.	ECM	1.73
10.15156/BIO/SH200219.07FU	JN871206	*Seimatosporium obtusum*	Pathogenic	1.38
10.15156/BIO/SH181879.07FU	AF444321	*Cryptococcus podzolicus*	Soil yeast	1.13
10.15156/BIO/SH189355.07FU	FJ876182	Thelephoraceae sp. 2	ECM/Saprotroph	1.05


*For ease of reading only those that have a greater than 1.00% relative abundance are listed with their taxonomic assignment, accession numbers and DOI.*

**FIGURE 5:**
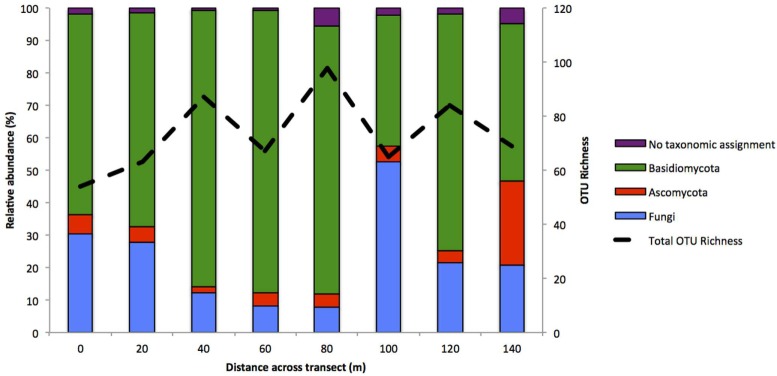
**Relative abundance of each phylum across the October 2010 transect produced via 454-pyrosequencing.** Additionally, the dashed line represents total OTU richness at each sampling location.

There was clear evidence of spatial variation within the root-associated fungal communities. Whilst Basidiomycota was the largest phylum within all samples, relative abundance ranged from a high of 87.1% within the community at 60 m across the transect, to a low of 40.1% within the site 100 m across (**Figure 5**). Ascomycota remained stable in relative abundance, ranging from 1.7 to 5.8% between sites 0–120 m. However, the Ascomycota accounted for 25.7% of the community at 140 m across the transect, largely driven by a 10.8% increase in reads assigned to a putative pathogen, *Seimatosporium*, which was absent in nearly all other samples. OTU richness also varied across transects, with the Basidiomycota ranging from 66 OTUs at the site 40m across the transect to 27 OTUs at 0m. Additionally, the Ascomycota ranged from just 4 OTUs at 40 m across the transect to 14 at 140 m. OTU richness within the Basidiomycota strongly strongly correlated (*R^2^* = 0.736, *P* = 0.037) with relative abundance but the Ascomycota did not (*R^2^* = 0.625, *P* = 0.097).

Average taxa richness produced via TRFLP for each sampling location was compared against OTU richness produced via 454-pyrosequencing (**Table 4**). Whilst 454-pyrosequencing produced significantly greater alpha diversity estimates for each sampling location (*t* = -5.161, *P* = 0.001), the trend in relative fungal richness was maintained between the two techniques, with a significant correlation of 0.767 (*P* < 0.05). Additionally, a DDR of -0.290 (*P* = 0.005) was calculated for the October 2010 root-associated fungal community analyzed with 454-pyrosequencing, and whilst this was higher than the DDR of -0.136 produced via TRFLP, there was no significant difference between the rates produced by differing techniques (*P* = 0.090).

**Table 4:** Comparison of TRF richness produced via TRFLP and OTU richness produced by 454-pyrosequencing for the October 2010 root-associated fungal communities.

Distance across transect (m)	TRFs	454-Pyrosequencing
0	44 (5)	54 (8)
20	22 (8)	63 (7)
40	68.3 (2)	87 (2)
60	30.8 (7)	67 (5)
80	83.3 (1)	98 (1)
100	34.8 (6)	65 (6)
120	46.8 (4)	84 (3)
140	62 (3)	69 (4)


*Brackets indicate alpha diversity site rank from highest to lowest fungal OTU richness.*

## Discussion

In this work, we show that both geographical distance and environmental variability (pH) simultaneously affect root-associated fungal community composition, with the magnitude of their influences being variable over time. A number of studies have found evidence for independent geographical distance effects on the composition of root-associated fungal communities (Green et al., 2004; Bahram et al., 2012; Põlme et al., 2013; Davison et al., 2015), whilst other studies have found no such effects (Tedersoo et al., 2012; Hazard et al., 2013; Mundra et al., 2015). However, here we suggest that distance effects plays a variable role in role in shaping the root-associated fungal community at the local level throughout time, whilst more temporal replicates are required to confirm if these effects are consistently greater later in the growing season within willow SRC plantations. CCAs revealed that 4.8% of community variation in October 2010 and 8.4% in October 2011 could be explained by geographical separation. Whilst CCAs revealed that the geographical separation between samples had a significant effect on the community compositions of the October 2010 and October 2011 transects, beta-diversity was significantly higher in October 2010, July 2011, and October 2011 than August 2011. Although the gradient in pH and soil nutrients affected the turnover of species over transects, these properties remained stable throughout the sampling period, suggesting factors other than edaphic properties affected the spatial turnover of species. Whilst this work was performed within a managed ecosystem, these findings are in agreement with a growing number of studies performed within unmanaged systems that suggest that the ubiquitous dispersal hypothesis does not hold true for fungi (Green et al., 2004; Põlme et al., 2013; Horn et al., 2014). Interestingly, studies which have not found distance effects on root-associated fungal community composition at the local and regional level have investigated this relationship within single sampling time points, and consequently may have missed the ‘window’ in time that geographical separation has an effect (Hazard et al., 2013; Põlme et al., 2013).

Our results suggest that the root-associated fungal community and the parameters that regulate community assembly vary throughout the year. Whilst effects of geographical distance on community assembly were variable throughout the year, in contrast pH played a near ubiquitous role in affecting community composition throughout the sampling period. When the temporal variation in the root-associated fungal community was analyzed, intra-annual variation (month of sampling) rather than inter-annual variation (time in weeks after sampling started) had the largest temporal effect on the community variation, suggesting the two October sampling points shared similarity to each other, and were distinct from the July and August assemblages. Time did, however, also independently affect root-associated fungal composition, indicating that longer-term processes such as ecological drift may also be occurring. Whilst there has been a number of previous studies demonstrating seasonal community dynamics, these have mostly been performed over single growing seasons and have not enabled year-on-year comparisons, therefore have been unable to isolate seasonal effects from other more long-term temporal processes (Jumpponen et al., 2010; Dumbrell et al., 2011; Martiny et al., 2011). In this work there was only 1 year-on-year comparison, therefore results should be interpreted cautiously. However, since the communities of October 2010 and 2011 shared the most similarity, yet were still significantly different, this suggests considerable complexity in the temporal variation of root-associated fungal communities, even within a relatively simple aboveground system. Whilst community shifts have been shown between sites differing in age by multiple years (Husband et al., 2002; Fujiyoshi et al., 2011; Blaalid et al., 2012), there is a need for further studies which define the relationships among inter-annual and intra-annual variations in fungal community assembly.

As within this study, ECM communities were found to vary globally with pH, but also with mean annual precipitation (Tedersoo et al., 2014). Changes in the composition of root-associated fungal communities over time could reflect either a direct response to annual variation in environmental parameters (such as precipitation) or indirect effects such as changing patterns of C supply from the plant. For example mature *Pinus sylvestris* was shown to increase plant derived carbon belowground by 500% later in the growing season compared to early growing season (Högberg et al., 2010), whilst the number of fine root tips of *Salix viminalis* has specifically been shown to increase throughout the growing season (Rytter and Hansson, 1996). Compositional shifts have also been observed in belowground microbial communities when belowground carbon allocation is increased (Phillips et al., 2002), suggesting changes in belowground plant growth dynamics throughout the year, in response to changing environmental conditions, may drive relatively short-term differences in fungal assemblages and their regulation found within this study. However, there are also a number of mechanisms that may not of been easily detected within the relatively short 13-month sampling period that may effect the root-associated fungal community. Long-term transitions in abiotic conditions, host age (Last et al., 1984; Visser, 1995; Husband et al., 2002), changing aboveground biomass composition (Blaalid et al., 2012) or even ecological succession series have been hypothesized to effect microbial assemblages over time (Deacon and Fleming, 1992; Bergemann and Miller, 2002) and there is a clear need for more studies contrasting inter and intra-annual variations within microbial communities.

454-pyrosequencing provides insightful taxonomic information about fungal composition and was used to confirm that a diverse root-associated fungal community resides within the willow monoculture. The PCR amplification with ITS1F and ITS4 primers successfully amplified a broad range of fungi and has been effectively used to detect Glomeromycota, Zygomycota, and Chytridiomycota in addition to Ascomycota and Basidiomycota within root-associated fungal communities (Yu et al., 2012), although only Ascomycota and Basidiomycota were detected within this study. These did, however, include ectomycorrhizal fungi, and a range of saprotrophs, endophytes and pathogenic fungi. Whilst many of the most abundant reads were assigned to probable ECM species, interestingly no reads were assigned to the Glomeromycota. *Salix* sp. can form ECM or AM associations depending on genotype and soil environment (Becklin et al., 2012; Corredor et al., 2012, 2014). Furthermore under some circumstances, AM inhibition by ECM fungi has been shown in laboratory experiments using willow, and this maybe replicated within willow plantations (Becklin et al., 2012).

TRFLP suffers from some well-documented limitations (Avis et al., 2006), therefore the pyrosequencing data from the October 2010 transect was also used to provide insight into the effectiveness of TRFLP to profile fungal communities. Whilst the fungal taxa richness produced via TRFLP was significantly lower than OTU richness produced via pyrosequencing, the trend in fungal richness and DDR across sampling locations with the two technologies was well conserved. This is in agreement with other studies that suggest microbial community profiles produced between TRFLP and pyrosequencing are reproducible (Pilloni et al., 2012). Pyrosequencing too suffers a number of limitations and the subsequent bioinformatics analyses can strongly impact taxa assignment, thus there is a great need to standardize high-throughput sequencing analyses in order to improve comparability between studies using the same as well as different technologies that profile soil communities (Tedersoo et al., 2015).

Given the importance of root-associated fungi in ecosystem functioning, understanding of the factors that regulate community assembly in environmental systems remain relatively unknown. Here we suggest that the spatial scaling of root-associated fungal communities does not follow the ubiquitous dispersal model, even at the local level, and the magnitude of this spatial scaling effect varies throughout the year. Future studies of root-associated fungal community assembly should therefore not underestimate the potential of distance effects occurring at the local geographic scale, and would also benefit from multiple sampling time points to fully characterize variation within ecosystems.

## Author Contributions

CB, main author of the manuscript, data producer and analysis. CvdG, assisted with statistical analyses and manuscript production. CAB, assisted in laboratory work and bioinformatics. NM, assisted in logistics, access to field sites and biogeochemical analyses. GB, main supervisor, obtained funding and initial experimental design.

## Conflict of Interest Statement

The authors declare that the research was conducted in the absence of any commercial or financial relationships that could be construed as a potential conflict of interest.
