# Supplementary material for: Temporally Variable Geographical Distance Effects Contribute to the Assembly of Root-Associated Fungal Communities
Source: Front Microbiol. 2016 Feb 25;7:195. doi: 10.3389/fmicb.2016.00195 (PMC4766365; doi:10.3389/fmicb.2016.00195)
Supplement: Supplementary file 1 [file Data_Sheet_1.DOCX]

## Barnes *et al*, Supporting Information

Article title: **TEMPORALLY VARIABLE GEOGRAPHICAL DISTANCE EFFECTS CONTRIBUTE TO THE ASSEMBLY OF ROOT-ASSOCIATED FUNGAL COMMUNITIES**

Authors: Christopher J. Barnes, Christopher J. van der Gast, Caitlin A. Burns, Niall P. McNamara, and Gary D. Bending

The following Supporting Information is available for this article:

Table S1 Table of *P*-values when comparing curves using the *t*-distribution method (Fowler *et al*, 1998).

|  | Oct-10 | Jul-11 | Aug-11 | Oct-11 |
| --- | --- | --- | --- | --- |
| Oct-10 | - | 0.921 | **<0.001** | 0.159 |
| Jul-11 |  | - | **<0.001** | 0.157 |
| Aug-11 |  |  | - | **<0.001** |
| Oct-11 |  |  |  | - |

Bold indicates significance (*P*<0.05).
